# An Empirical Study of Different Approaches for Protein Classification

**DOI:** 10.1155/2014/236717

**Published:** 2014-06-15

**Authors:** Loris Nanni, Alessandra Lumini, Sheryl Brahnam

**Affiliations:** ^1^Dipartimento di Ingegneria dell'Informazione, Via Gradenigo 6/A, 35131 Padova, Italy; ^2^DISI, Università di Bologna, Via Venezia 52, 47521 Cesena, Italy; ^3^Computer Information Systems, Missouri State University, 901 South National, Springfield, MO 65804, USA

## Abstract

Many domains would benefit from reliable and efficient systems for automatic protein classification. An area of particular interest in recent studies on automatic protein classification is the exploration of new methods for extracting features from a protein that work well for specific problems. These methods, however, are not generalizable and have proven useful in only a few domains. Our goal is to evaluate several feature extraction approaches for representing proteins by testing them across multiple datasets. Different types of protein representations are evaluated: those starting from the position specific scoring matrix of the proteins (PSSM), those derived from the amino-acid sequence, two matrix representations, and features taken from the 3D tertiary structure of the protein. We also test new variants of proteins descriptors. We develop our system experimentally by comparing and combining different descriptors taken from the protein representations. Each descriptor is used to train a separate support vector machine (SVM), and the results are combined by sum rule. Some stand-alone descriptors work well on some datasets but not on others. Through fusion, the different descriptors provide a performance that works well across all tested datasets, in some cases performing better than the state-of-the-art.

## 1. Introduction

The explosion of protein sequences generated in the postgenomic era has not been followed by an equal increase in the knowledge of protein biological attributes, which are essential for basic research and drug development. Since manual classification of proteins by means of biological experiments is both time-consuming and costly, much effort has been applied to the problem of automating this process using various machine learning algorithms and computational tools for fast and effective classification of proteins given their sequence information [1]. According to [2], a process designed to predict an attribute of a protein based on its sequence generally involves the following procedures: (1) constructing a benchmark dataset for testing and training machine learning predictors, (2) formulating a protein representation based on a discrete numerical model that is correlated with the attribute to predict, (3) proposing a powerful machine learning approach to perform the prediction, (4) evaluating the accuracy of the method according to a fair testing protocol, and (5) establishing a user-friendly web-server accessible to the public.

In this work we are mainly interested in the second procedure, that is, in the definition of a discrete numerical representation for a protein. Since many different representations have been proposed in the literature, it would be valuable to investigate which of these are most useful for the specific applications, such as subcellular localization and protein-protein interactions [3–6], to which these representations are applied [7, 8].

Two kinds of models are typically employed to represent protein samples: the sequential model and the discrete model. The most widely used sequential model is based on the entire amino-acid sequence of a protein, expressed by the sequence of its residues, with each one belonging to one of the 20 native amino-acid types:

(1) $$\begin{array}{c} P=\left(p_{1},p_{2},\ldots ,p_{N}\right)\;\text{where}\;p_{i}\in A=\left[\text{A},\text{C},\text{D},\ldots ,\text{Y}\right]. \end{array}$$

This kind of approach, whose length varies depending on the protein structure, is not suited for most machine learning predictors and fails to work when the query protein does not have significant sequence similarity to any attribute-known proteins.

More suitable for machine learning purposes are protein discrete models, which fall into two main classes. The first class includes the simple amino-acid composition (AAC) and approaches that are based on the AAC-discrete model, such as Chou's pseudo-amino-acid composition (PseAAC) [3–5], which is arguably one of the most popular methods for extracting features from proteins. This first class includes techniques based on vector representations of the protein, that is, where a protein sequence *P* = (*p*_1_, *p*_2_,…, *p*_*N*_) is represented by a vector ∈*R* ^N^. In [9] AAC is a vector of length 20 that includes the normalized occurrence frequencies of the 20 native amino acids. PseAAC [10, 11] expands AAC by retaining information embedded in protein sequences, such as some additional factors that incorporate information regarding a protein's sequential order. Various modes, such as a series of rank-different correlation factors along a protein chain, represent the sequential information. For an excellent history of the development of PseAAC that includes how to use the concept of Chou's PseAAC to develop 16 variant forms of PseAAC, the reader is referred to [11]. In [12] a protein representation based on physicochemical encodings is proposed that combines the value of a given property for an amino acid with its 2 grams representation. Another vector representation is the quasiresidue couple [13], a model which combines information related to a fixed physicochemical property of the protein with the sequence order effect of the composition of the amino acid. Other approaches belonging to this class of protein representation include dipeptide [14], tripeptide [15], and tetrapeptide [16]. These approaches are based on *n*-peptide descriptors, where each protein is represented by a vector of length 20^*n*^ that includes the normalized occurrence frequencies of the given *n*-peptide. For reducing the dimensionality of the descriptor, a feature selection algorithm may be used, as in [16].

Before proceeding to the second class of representations based on protein discrete models, it should be noted that a number of different PseAAC methods have been developed for specific applications, such as for predicting certain biological attributes. Some examples include cellular automata image classification [17–20], complexity measure factor [19, 21], gray dynamic model [17, 18], and functional domain composition [20].

The second class of protein feature extraction methods is based on kernels. One of the first kernel-based methods (proposed for remote homology detection) is the Fisher kernel [22]. A kernel that performs equally well but with lower computational cost is the mismatch string kernel proposed in [23, 24] that measures sequence similarity based on shared occurrences of subsequences. In [25] another class of kernels is proposed for vectors derived from the k-peptide vector, mapped by a matrix of high-scored pairs of k-peptides measured by BLOSUM62 scores. These kernel functions train a support vector machine (SVM). In [26] the biobasis function neural network trains sequence distances obtained using sequence alignment.

Aside from using AAC and protein properties for protein representation, several high performing features have also been derived from the position-specific scoring matrix (PSSM) [27]. PSSM describes a protein starting from the evolutionary information contained in a PSI-BLAST similarity search. For a survey of research using descriptors extracted from PSSM, see [28].

The main drawback of the methods based on structural or sequential features is that they only focus on the local variation of the protein itself. For this reason, cellular interactions of proteins have been investigated, as in [29], for solving some particular problems. In [30], the combination of traditional sequential features of the amino acid, such as PSSM, and different networks, such as KEGG enrichment scores of the protein neighbors in STRING network [31], were studied. Protein interaction networks have also been examined in [32, 33].

In this study our objective is to search for a general ensemble method that works well across different protein classification datasets. To accomplish our goal we focus on structural and sequential features. We are motivated to study protein classification methods that generalize well because such systems offer the potential of deepening our understanding of protein representation and of speeding up real world development in new areas involving protein classification. Such investigations also have the potential of promoting and laying the foundations for the development of more robust and powerful classification systems.

The present paper provides an in-depth look at the protein representations that have led to the evolution of some of our previous work in this area.

- Reference [12], where an ensemble of approaches based on the amino-acid sequence was proposed.
- Reference [34], where several feature extraction methods based on the calculation of texture descriptors starting from a wavelet representation of the protein were proposed.
- Reference [28], where several feature extraction methods based on the calculation of PSSM were compared.

In this work we explain and compare several state-of-the-art descriptors and some new variants starting from different types of protein representations: the PSSM, the amino-acid sequence, two matrix representations of the protein, and the 3D tertiary structure representations of the protein. We also develop a new ensemble (based on the above cited works) that performs well across multiple datasets, with our ensemble obtaining state-of-the-art performances on several datasets. For the sake of fairness, we use the same ensemble with the same set of parameters (i.e., the same weights in the weighted sum rule) across all tested datasets.

The remainder of this paper is organized as follows. In Section 2 the entire ensemble system is described, including all the protein representation approaches and feature extraction methods we use, all of which are detailed in Sections 3 and 4, respectively. In Section 5 the datasets used for experiments are described, and the results of several experiments conducted both with stand-alone approaches and ensembles of methods are reported and discussed. Finally, in Section 6, a number of conclusions are drawn and some future lines of research are proposed.

## 2. A General Machine Learning Approach for Protein Classification

Since several problems in the bioinformatics literature require the classification of proteins, a number of datasets are available for experiments, and recent research has focused on finding a compact and effective representation of proteins [3–5, 12], possibly based on a fixed-length descriptor so that the classification problem can be solved by a machine learning approach. In this work several solutions are evaluated based on a general representation approach that is coupled with a fixed-length encoding scheme so that it can be used with a general purpose classifier.

**Figure 1:**
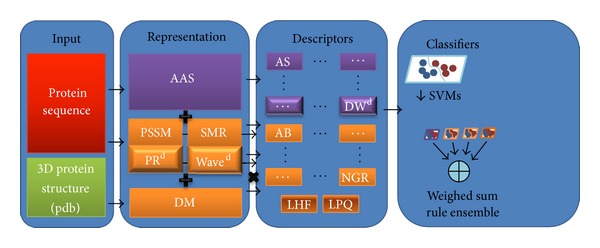
Schema of the proposed method.

The classification system illustrated in Figure 1 is an ensemble of classifiers trained using the different descriptors. Five types of protein representations are considered for all the datasets: the simple amino-acid sequence (AAS), PSSM of the proteins, substitution matrix representation (SMR), physicochemical property response matrix (PR), and the wavelet image (WAVE). A detailed description of each representation is given in Section 3. From each representation several descriptors are extracted, which we describe in Section 4. Some descriptors are extracted multiple times, once for each physicochemical property considered in the extraction process. The set of physicochemical properties is obtained from the amino-acid index [35] database (available at http://www.genome.jp/dbget/aaindex.html but note that we do not consider properties where the amino acids have value 0 or 1). An amino-acid index is a set of 20 numerical values representing the different physicochemical properties of amino acids. This database currently contains 544 indices and 94 substitution matrices, but a reduced number of properties are enough for classification task. According to [12], a selection of 25 properties is performed to reduce the number of properties considered in the feature extraction process.

The combination of representation and descriptors is summarized in Table 1, with the size of each descriptor reported in Table 1.

Each descriptor is used to train a general purpose classifier. SVMs are used for the classification task due to their wide diffusion and high generalization ability. SVMs derive from the field of statistical learning theory [36] and are binary-class prediction methods. The basic idea behind SVMs is to find the equation of a hyperplane (called the margin) that divides the training set into two classes so that all the points of the same class are located on the same side while simultaneously maximizing the distance between the two classes and the margin. In those problems where a linear decision boundary does not exist, kernel functions are used to project the data onto a higher-dimensional feature space so that they can be separated by a hyperplane. Typical kernels include polynomial kernels and the radial basis function kernel. SVMs can be easily extended to multiclass problems by considering the one-versus-all classification task. In the experiments reported in this work, all features used for training an SVM are linearly normalized to [0,1] considering the training data. In each dataset the SVM is tuned considering only the training data (in other words, the test is blind) using a grid search approach. In our system, SVM is implemented as in the LibSVM toolbox (available at http://www.csie.ntu.edu.tw/?cjlin/libsvm).

The ensemble approaches based on the fusion of different descriptors are obtained by combining the pool of SVMs by weighted sum rule; this rule simply sums the scores obtained by the pool of SVMs classifiers, where to each SVM a given weight is applied.

## 3. Protein Representation Approaches

### 3.1. A Sequential Representation for Proteins: Amino-Acid Sequence (AAS)

The most widely used representation for proteins is a sequential model of the amino-acid sequence:

(2) $$\begin{array}{c} P=\left(p_{1},p_{2},\ldots ,p_{N}\right), \end{array}$$

where *p*_*i*_ ∈ *A* = [A, C, D,…, Y] and *A* is the set of the 20 native amino-acid types. Several studies [35] have shown that AAS coupled with other information related to the physicochemical properties of amino acids produces many useful descriptors, some of which will be described in Section 4.

### 3.2. A Matrix Representation for Proteins: Position-Specific Scoring Matrix (PSSM)

The PSSM representation of a protein, first proposed in [27], is obtained from a group of sequences previously aligned by structural or sequence similarity. Such representations can be calculated using the application PSI-BLAST (position-specific iterated BLAST), which compares PSSM profiles for detecting remotely related homologous proteins or DNA.

The PSSM representation considers the following parameters.

1. Position: the index of each amino-acid residue in a sequence after multiple sequence alignment.
2. Probe: a group of typical sequences of functionally related proteins already aligned by sequence or structural similarity.
3. Profile: a matrix of 20 columns corresponding to the 20 amino acids.
4. Consensus: the sequence of amino-acid residues most similar to all the alignment residues of probes at each position. The consensus sequence is generated by selecting the highest score in the profile at each position.

A PSSM representation for a given protein of length *N* is an *N* × 20 matrix, whose elements PSSM(*i*, *j*) are calculated as

(3) PSSM(i,j)=∑k=120w(i,k)×Y(j,k),i=1,…,N, j=1,…,20,

where *w*(*i*, *k*) is the ratio between the frequency of the *k*th amino acid at the position *i* of the probe and total number of probes and *Y*(*j*, *k*) is the value of Dayhoff's mutation matrix between the *j*th and *k*th amino acids (*Y*(*j*, *k*) is a substitution matrix). A substitution matrix describes the rate at which one character in a protein sequence changes to other character states over time. Substitution matrices are usually seen in the context of amino acid or DNA sequence alignments, where the similarity between sequences depends on their divergence time and the substitution rates as represented in the matrix.

Small values of PSSM(*i*, *j*) indicate weakly conserved positions and large values indicate strongly conserved positions. In our study, we used PSI-BLAST which can be called from MATLAB for extracting PSSM using the command system (“blastpgp.exe-i input.txt-d swissprot-Q output.txt-j 3,” where “input.txt” is the protein sequence and “output.txt” contains the PSSM matrix to create PSSMs for each protein sequence).

### 3.3. A Matrix Representation for Proteins: Substitution Matrix Representation (SMR)

In [28] a variant of a representation method called the substitution matrix representation (SMR) proposed by [37] is developed where the SMR for a given protein *P* = (*p*_1_, *p*_2_,…, *p*_*N*_) is a *N* × 20 matrix obtained as

(4) $$\begin{array}{c} {\text{SMR}}^{d}\left(i,j\right)=M\left(p_{i},j\right),\;i=1,\ldots ,N,\;\;j=1,\ldots ,20, \end{array}$$

where *M*(*a*, *j*) is a 20 × 20 substitution matrix whose element *M*_*a*,*j*_ represents the probability of amino acid *a* mutating to amino acid *j* during the evolution process (note: the MATLAB code for this representation is available at http://bias.csr.unibo.it/nanni/SMR.rar).

In the experiments reported below, 25 random physicochemical properties have been selected to create an ensemble (labelled SMR) of SMR^*d*^-based predictors.

### 3.4. A Matrix Representation for Proteins: Physicochemical Property Response Matrix (PR)

In [34] a representation matrix based on physicochemical properties is proposed. First the physicochemical property response matrix PRM^*d*^(*i*, *j*) ∈ *R* ^*N*×*N*^ is obtained for a given protein *P* = (*p*_1_, *p*_2_,…, *p*_*N*_) by selecting a physicochemical property *d* and setting the value of the element PRM^*d*^(*i*, *j*) to the sum of the value of the physicochemical property *d* of the amino-acid in position *i* of the protein and the value of the physicochemical property of the amino-acid in position *j*. Consider

(5) PRMd(i,j)=index(pi,d)+index(pj,d),i,j=1,…,N,

where index(*a*, *d*) returns the value of the property *d* for the amino acid *a*.

Then PRM^*d*^ is handled as an image and resized to 250 × 250 if larger to obtain the final matrix PR^*d*^. In the experiments reported below, 25 random physicochemical properties have been selected to create an ensemble (PR) of PR^*d*^-based predictors.

### 3.5. A Matrix Representation for Proteins: Wavelet (WAVE)

Wavelets are very useful descriptors with lots of different applications. First [38] then later [34] have suggested using wavelets as a method to represents proteins. Since wavelet encoding requires a numerical representation, the protein sequence is first numerically encoded by substituting each amino acid with a value of a given physicochemical property *d*. Then the Meyer continuous wavelet is applied to the wavelet transform coefficients (labelled WAVE^*d*^). Features are extracted by considering 100 decomposition scales. In the experiments reported below, 25 random physicochemical properties have been selected to create an ensemble WAVE of 25 WAVE^*d*^-based predictors.

### 3.6. A Matrix Representation for Proteins: 3D Tertiary Structure (DM)

The 3D tertiary structure representation for proteins is based on the protein backbone (i.e., the sequence of its *C*_*α*_ atoms) to characterize the whole protein structure [39, 40]. Given a protein *P* and its backbone *B* = (**C** **o** **o** **r**_1_, **C** **o** **o** **r**_2_,…, **C** **o** **o** **r**_*M*_) (obtained as the 3D coordinates of its *M* *C*_*α*_ atoms), a distance matrix DM is defined as

(6) $$\begin{array}{c} \text{DM}\left(i,j\right)=\mathrm{dist}\left({Coor}_{i},{Coor}_{j}\right),\;1\leq i,\;\;j\leq M, \end{array}$$

where dist⁡(·) is the Euclidean distance. (Note: the MATLAB code for extracting the distance matrix is available at http://bias.csr.unibo.it/nanni/DM.zip).

**Figure 2:**
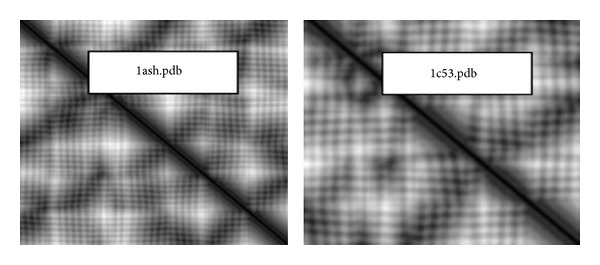
DM images extracted from 2 sample proteins of the DNA dataset.

As with the other matrix representations introduced above, DM is regarded as a grayscale image, which is used to extract texture descriptors, as illustrated in Figure 2.

## 4. Protein Feature Extraction Approaches

In this section the approaches used to extract descriptors from the different representations introduced above are described. Most of the descriptors extracted from the primary representation are based on substituting the letter representation of an amino acid with its value of a fixed physicochemical property. In order to make the result independent on the selected property, a selection on 25 or 50 properties is done by random, and the resulting descriptors are used to train an ensemble of SVM classifiers.

### 4.1. A Descriptor for Primary Representation: Amino-Acid Composition (AS)

Amino-acid composition is the simpler method for extracting features from a protein representation that is based on counting the fraction of a given amino acid:

(7) $$\begin{array}{c} \text{AS}\left(i\right)=\frac{h\left(i\right)}{N},\;i\in \left[1,\ldots ,20\right], \end{array}$$

where *h*(*i*) counts the number of occurrences of a given amino acid in a protein sequence of length *N*.

### 4.2. A Descriptor for Primary Representation: 2 Grams (2G)

The standard 2 grams descriptor is a vector of 20^2^ values, each counting the number of occurrences of a given couple of amino acids in a protein sequence. Consider

(8) $$\begin{array}{c} 2\text{G}\left(k\right)=\left(\frac{h\left(i,j\right)}{N}\right),\;i,j\in \left[1,\ldots ,20\right]\text{,}\;\;k=j+20\times \left(i-1\right), \end{array}$$

where the function *h*(*i*, *j*) counts the number of occurrences of a given couple of amino acids (*i*, *j*) in a protein sequence of length *N*. The 2G descriptor is a 400-dimensional vector.

### 4.3. A Descriptor for Primary Representation: Quasiresidue Couple (QRC)

Quasiresidue Couple is a method for extracting features from the primary sequence of a protein [12] that is inspired by Chou's quasi-sequence-order model and Yuan's Markov chain model [41]. The original residue couple model was designed to represent both the information of the amino-acid composition and the order of the amino acids in the protein sequences. The quasiresidue couple descriptor is obtained by selecting a physicochemical property *d* and combining its values with each nonzero entry in the residue couple. The parameter *m* denotes the order of the composition (values *m* ≤ 3 are considered enough to represent a sequence).

The QRC model (of order *m* ≤ 3) for a physicochemical property *d* is given by

(9) QRCmd(k)=1N−m∑n=1N−mHi,j(n,n+m,d)+Hj,i(n+m,n,d),i,j∈[1,…,20], k=j+20(i−1),

where *i* and *j* are the 20 different amino acids, *N* is the length of the protein, the function index(*i*, *d*) returns the value of the property *d* for the amino acid *i*, and the function *H*_*i*,*j*_(*a*, *b*, *d*) = index(*i*, *d*), if *p*_*a*_ = *i* and *p*_*b*_ = *j*, *H*_*i*,*j*_(*a*, *b*, *d*) = 0 otherwise.

In our experiments, the QRC^*d*^ features are extracted for *m* ranging from 1 to 3 and concatenated into a 1200-dimensional vector. In the experiments reported below, 25 random physicochemical properties have been selected to create an ensemble of QRC descriptors (Note: the MATLAB code for QRC is available at http://bias.csr.unibo.it/nanni/QRcouple2.zip).

### 4.4. A Descriptor for Primary Representation: Autocovariance Approach (AC)

The autocovariance approach [42] is a sequence-based variant of Chou's pseudo-amino-acid composition, which extracts a set of pseudo-amino-acid-based features (extracted by the MATLAB code shared by the original authors) from a given protein as the concatenation of the 20 standard amino-acid composition values and *m* values reflecting the effect of sequence order. The parameter *m* denotes the maximum distance between two considered amino acids *i*, *j* (set to 20 in the tests reported below).

Given a protein *P* = (*p*_1_, *p*_2_,…, *p*_*N*_) and fixing a physicochemical property *d*, the autocovariance descriptor is AC⁡^*d*^ ∈ *R* ^20+*m*^:

(10) AC⁡d(i) ={h(i)N, i∈[1,…,20],∑k=1N−i+20(index(pk,d)−μd)·(index(pk+i−20,d)−μd)σd·(N−i+20), i∈[21,…,20+m],

where the function index(*i*, *d*) returns the value of the property *d* for the amino acid *i*, the function *h*(*i*) counts the number of occurrences of a given amino acid in a protein sequence, and *μ*_*d*_ and *σ*_*d*_ are normalization factors denoting mean and the variance of *d* on the 20 amino acids:

(11) $$\begin{array}{c} \mu_{d}=\frac{1}{20}\overset{20}{\underset{i=1}{\sum }}\text{index}\left(i,d\right),\\ \sigma_{d}=\frac{1}{20}\overset{20}{\underset{i=1}{\sum }}{\left(\text{index}\left(i,d\right)-\mu_{d}\right)}^{2}. \end{array}$$

In the experiments reported below, 25 random physicochemical properties have been selected to create an ensemble of 25 AC descriptors (Note: the MATLAB code for AC is available at http://bias.csr.unibo.it/nanni/EstraggoFeaturesAC.rar).

### 4.5. A Descriptor for Primary Representation: AAIndexLoc (AA)

The AAIndexLoc is a descriptor proposed in [43]. AAIndexLoc is composed of the following features.

- *Amino-acid composition (20 features)*: this is a fraction of a given amino acid.
- *Weighted amino-acid composition (20 features)*: this is defined for a given amino acid *i* as (amino-acid composition of *i*) × index(*i*, *d*).
- *Five-level grouping composition (25 features)*: the result of a five-level dipeptide composition applied to a classification of the amino acids by k-means (into five groups) considering their amino-acid index values. The five-level dipeptide composition is defined as the composition of the occurrence of two consecutive groups; see [43] for more details.

In the experiments reported below, 25 random physicochemical properties have been selected to create an ensemble of 25 AA^*d*^ descriptors.

### 4.6. A Descriptor for Primary Representation: Global Encoding (GE)

Global encoding is a descriptor proposed in [44] that is based on a classification (labelled here as A) of amino acids into six classes: A1 = {A, V, L, I, M, C}, A2 = {F, W, Y, H}, A3 = {S, T, N, Q}, A4 = {K, R}, A5 = {D, E}, and A6 = {G, P}. The final descriptor GE is obtained by extracting measures from the 10* characteristic sequences, *CS_Pt_, obtained from each of the 10 different* partitions* Pt of A into 2 subsets of three classes (e.g., Pt = {(A1, A2, A3), (A4, A5, A6)} is one of the 10 partitions). CS_Pt_ is obtained by transforming the protein into a numerical sequence where a given amino acid is represented by 1 if it belongs to the first class of the partition (i.e., (A1, A2, A3)) and 0 otherwise. The two sets of measures used to define GE are the frequency of 0s and 1s in each CS_Pt_ and the frequency of transitions (i.e., number of 1s followed by a 0, and vice versa).

### 4.7. Descriptor for Primary Representation: Physicochemical 2 Grams (P2G)

The physicochemical 2 grams [13] are descriptors that combine the value of a given physicochemical property *d* for an amino acid together with the 2 grams representation of a protein. The standard 2 grams representation is a vector of 20^2^ values, each counting the number of occurrences of a given couple of amino acids in a protein sequence. The physicochemical 2 grams (P2G) for a given physiochemical property *d* is defined as

(12) P2Gd(k)=(h(i,j)·index(i,d)N−1,h(i,j)·index(j,d)N−1),i,j∈[1,…,20], k=j+20(i−1),

where *i* and *j* denote the 20 different amino acids, *N* is the length of the protein, the function index(*i*, *d*) returns the value of the property *d* for the amino acid *I*, and the function *h*(*i*, *j*) counts the number of occurrences of a given couple of amino acids (*i*, *j*) in a protein sequence. The P2G^*d*^ descriptor is an 800-dimensional vector. In the experiments reported below, 25 random physicochemical properties have been selected to create an ensemble of 25 P2G^*d*^ descriptors.

### 4.8. A Descriptor for Primary Representation: N-Gram (NG)

The N-gram descriptor is similar to the standard 2 grams descriptor but is obtained on a different *N*-peptide composition using different amino-acid alphabets. In this work 5 alphabets proposed in [45] are considered, and we train five different SVMs. Each classifier is trained using a different *N*-peptide composition with different amino-acid alphabets:

- A1 = G–I–V–F–Y–W–A–L–M–E–Q–R–K–P–N–D–H–S–T–C,
- A2 = LVIM–C–A–G–S–T–P–FY–W–E–D–N–Q–KR–H,
- A3 = LVIMC–AG–ST–P–FYW–EDNQ–KR–H,
- A4 = LVIMC–ASGTP–FYW–EDNQ–KRH,
- A5 = LVIMC–ASGTP–FYW–EDNQKRH.

Each protein is first translated according to the 5 alphabets. Then 2 gram representations are calculated from A1 to A2 languages, and the 3 gram representations are calculated from A3 to A4 to A5. The five descriptors are

(13) $$\begin{array}{c} {\text{NG}}^{A}\;A\in \left[\text{A}1,\text{A}2,\text{A}3,\text{A}4,\text{A}5\right], \end{array}$$

having dimensions #*A* ^*n*^, where *n* = 2 for 2 gram representations and *n* = 3 for 3 gram representations. The 5 representations are fused together by weighted sum rule (with weights 1, 1, 1, 0.5, and 0.25).

### 4.9. A Descriptor for Primary Representation: Split Amino-Acid Composition (SAC)

Split amino-acid composition is a descriptor proposed by [46] that is based on the subdivision of the protein sequence into parts from which a separate descriptor is calculated (i.e., the standard amino-acid composition for each part). In this work each protein is divided into the following three parts: (i) 20 amino acids of N-terminus, (ii) 20 amino acids of C-terminus, and (iii) the region between these two termini.

### 4.10. A Descriptor for Primary Representation: Discrete Wavelet (DW)

A sequence descriptor based on biorthogonal discrete wavelet is proposed in [34]. Given a protein *P* = (*p*_1_, *p*_2_,…, *p*_*N*_) and a fixed physicochemical property *d*, each amino acid of the sequence is substituted by its value of *d*:

(14) $$\begin{array}{c} {\text{PP}}^{d}\left(i\right)=\text{index}\left(p_{i},d\right),\;i\in \left[1,\ldots ,N\right], \end{array}$$

where the function index(*i*, *d*) returns the value of the property *d* for the amino acid *i*.

The vector PP^*d*^ is then transformed in the wavelet space by a four-scale biorthogonal discrete wavelet. The final descriptor DW^*d*^ is obtained as the first five discrete cosine coefficients from the approximation coefficients and the maximum, minimum, mean, and standard deviation values from both detail and approximation coefficients. This choice is motivated by the fact that high-frequency components are noisy; thus the low-frequency components are more useful for the classification task.

In the experiments reported below, 25 random physicochemical properties have been selected to create an ensemble of 25 DW^*d*^ descriptors.

### 4.11. A Matrix-Based Descriptor: Average Blocks (AB)

This matrix descriptor was originally proposed in [47] for the PSSM representation of a protein, but it is used for other matrix representations in this work. Average blocks is a fixed-length vector AB ∈ *R* ^400^ elements obtained as the local average of the input matrix Mat ∈ *R* ^*n*×20^:

(15) AB(k)=20N∑z=1N/20Mat(z+(i−1)∗N20,j),i=1,…,20, j=1,…,20, k=j+20×(i−1),

where *k* is a linear index used to scan the cells of Mat. Thus the final descriptor is a vector obtained as the average of Mat blocks (each related to the 5% of a sequence).

### 4.12. A Matrix-Based Descriptor: Single Average (SA)

This descriptor [48] is a variant of the previous one and is designed to group together rows related to the same amino acid, thus considering domains of a sequence with similar conservation rates.

The descriptors SA ∈ *R* ^400^ for a protein *P* = (*p*_1_, *p*_2_,…, *p*_*N*_) and its matrix representation Mat is

(16) SA(k)=avgi=1,…,N⁡Mat(i,j)∗δ(P(i),A(z)),z=1,…,20, j=1,…,20, k=j+20×(z−1),

where *k* is a linear index used to scan the cells of Mat, where *A* = [A, C, D,…, Y] is the ordered set of amino acids, and where *δ*(·, ·) is the Kronecker delta function.

In this work two variants of the single average descriptor are used: the one described above (labelled SA) and a version including matrix normalization using a sigmoid function by which each element of Mat is scaled to [0,1] (labelled SAN).

### 4.13. A Matrix-Based Descriptor: Autocovariance Matrix (AM)

The autocovariance matrix is a matrix descriptor proposed in [49] that aims at avoiding the loss of the local sequence-order information. Each column of the input matrix is reduced to a fixed length by autocovariance variables. An autocovariance matrix (AM) describes the average correlation between positions in a series of lags (i.e., the residue number when applied to protein sequences) throughout the protein sequence.

AM can be calculated from an input matrix Mat ∈ *R* ^*N*×20^ as follows:

(17) AM(k)=1N−lag∑i=1N−lag(Mat(i,j)−1N∑i=1NMat(i,j))     ×(Mat(i+lag,j)−1N∑i=1NMat(i,j)),j=1,…,20, lag=1,…,15, k=j+20×(lag−1),

where *k* is a linear index used to scan the cells of Mat, lag denotes the distance between one residue and its neighbors, and *N* is the length of the sequence.

### 4.14. A Matrix-Based Descriptor: Pseudo-PSSM (PP)

This pseudo-PSSM approach (PP) is one of the most widely used matrix descriptors for proteins (see [47, 50]). Usually applied to the PSSM matrix representation of a protein, PP is extended in this work to SMR. This descriptor is designed to retain information about amino-acid sequence by considering the pseudo-amino-acid composition.

Given an input matrix Mat ∈ *R* ^*N*×20^, the pseudo-PSSM descriptor is a vector PP ∈ *R* ^320^ defined as

(18)  PP(k)={1N∑i=1NE(i,j), k=1,…,20,1N−lag∑i=1N−lag[E(i,j)−E(i+lag,j)]2, j=1,…,20,  lag=1,…,15, k=20+j+20·(lag−1),

where *k* is a linear index used to scan the cells of Mat, where lag denotes the distance between one residue and its neighbors, and where *N* is the length of the sequence and *E* ∈ *R* ^*N*×20^, which is a normalized version of Mat defined as

(19) E(i,j)=Mat(i,j)−(1/20)∑v=120Mat(i,v)(1/20)∑u=120(Mat(i,u)−(1/20)∑v=120Mat(i,v))2,i=1,…,N, j=1,…,20.

### 4.15. A Matrix-Based Descriptor: Singular Value Decomposition (SVD)

Singular value decomposition is a general purpose matrix factorization approach [51] that has many useful applications in signal processing and statistics. In this work SVD is applied to a matrix representation of a protein with the aim of reducing its dimensionality.

Given an input matrix Mat ∈ *R* ^*N*×*M*^, SVD is used to calculate its factorization of the form: Mat = *U*Σ*V*, where Σ is a diagonal matrix whose diagonal entries are known as the singular values of Mat. The resulting descriptor is the ordered set of singular values: SVD ∈ *R* ^*L*^, where *L* = min⁡{*M*, *N*}.

### 4.16. A Matrix-Based Descriptor: Discrete Cosine Transform (DCT)

DCT [52] is a linear separable transformation for converting a signal into elementary frequency components; it is widely used in image compression for its capability to concentrate information into a small number of coefficients. Given an input matrix Mat ∈ *R* ^*N*×*M*^, its DCT transformation is defined as

(20) DCT(i,j)=aiaj∑m=0M−1 ∑n=0N−1Mat(m,n)cos⁡π(2m+1)i2M      ×cos⁡π(2n+1)i2N,    0≤i≤M, 0≤j≤N,

where

(21) $$\begin{array}{c} a_{i}=\{\begin{array}{cc} \frac{1}{\sqrt{M}} & i=0,\\ \sqrt{\frac{2}{M}} & 1\leq i\leq M-1, \end{array}\\ a_{i}=\{\begin{array}{cc} \frac{1}{\sqrt{N}} & j=0,\\ \sqrt{\frac{2}{N}} & 1\leq j\leq N-1. \end{array} \end{array}$$

In this work the final DCT descriptor is obtained by retaining the first 400 coefficients.

### 4.17. A Matrix-Based Descriptor: N-Gram Features (NGR)

The N-gram descriptor is usually extracted from the primary protein sequence (as already described in Section 4.6). In [53] this descriptor is extracted directly from the PSSM matrix by accumulating the probabilities of each of the N-gram according to the probability information contained in PSSM.

Given an input matrix Mat ∈ *R* ^*N*×20^ representing the PSSM of a given protein, the frequency of occurrence of transition from *i*th amino acid to *j*th amino acid is calculated as follows for 2 grams (BGR) and 3 grams (TGR), respectively:

(22) BGR(l)=∑z=1N−1Mat(z,i)×Mat(z+1,j),i=1,…,20, j=1,…,20, l=(i−1)∗20+j,TGR(l)=∑z=1N−2Mat(z,i)×Mat(z+1,j)×Mat(z+2,k)i=1,…,20, j=1,…,20, k=1,…,20,l=(i−1)∗400+(j−1)∗20+k.

### 4.18. A Matrix-Based Descriptor: Texture Descriptors

A very interesting feature extraction approach for proteins is to treat a protein matrix representation as an image and to use well-known image texture descriptors for extracting features. In this work two high performing descriptors are evaluated: local binary pattern histogram fourier (LHF) descriptors [54] and local phase quantization (LPQ) (Note: the MATLAB code for LBQ is available at http://www.cse.oulu.fi/CMV/Downloads/LPQMatlab) [55]. Both these descriptors are extracted according to a global and a local evaluation (i.e., from the whole image or from subwindows of an image with the results of each concatenated). The feature vectors extracted are labelled, respectively: LHF_G and LPQ_G, when extracted from the whole image, and LHF_L and LPQ_L, when obtained by dividing the image into three equal subwindows and concatenating the resulting feature vectors.

#### 4.18.1. Local Binary Pattern Histogram Fourier (LHF)

First proposed by [54], LHF is a rotation invariant image descriptor that is computed from the discrete Fourier transforms of local binary pattern (LBP) histograms. The LHF descriptor computes a noninvariant LBP histogram and constructs rotationally invariant features from the histogram using discrete Fourier transform. The features are invariant to cyclic shifts in the input vector. In this work the final vector is the concatenation of results obtained using the following parameters for LBP computation: (*P* = 16; *R* = 2) and (*P* = 8; *R* = 1).

#### 4.18.2. Local Phase Quantization (LPQ)

The LPQ operator [55] is based on the blur invariance property of the Fourier phase spectrum. LPQ uses the local phase information extracted from the two-dimensional short-term Fourier transform (STFT) computed over a rectangular neighborhood defined by each pixel position. After STFT only four complex coefficients are retained corresponding to four fixed two-dimensional frequencies, which are separated into real and imaginary parts and quantized as integers between 0–255 using a binary coding scheme. The final feature vector is a normalized histogram of such coefficients. In this work the final vector is the concatenation of results obtained with two different radii for LPQ computation: radii 3 and 5, using the Gaussian derivative quadrature filter pair for local frequency estimation (Note: we used the MATLAB code for LPQ available at http://www.ee.oulu.fi/mvg/download/lpq/).

## 5. Experiments

This section reports the results of an experimental evaluation of the protein descriptors on sequence-based protein classification problems performed on several datasets.

### 5.1. Datasets, Testing Protocols, and Performance Indicators

The proposed approach has been evaluated on the 15 datasets listed below and according to the testing protocols suggested by the developers of the datasets. A brief summary description of each dataset and related testing protocol is reported in Table 2.

*Membrane Subcellular (MEM) (See [56])*. This is a dataset containing membrane proteins belonging to eight membrane types: (1) single-pass type I transmembrane, (2) single-pass type II, (3) single-pass type III, (4) single-pass type IV, (5) multipass transmembrane, (6) lipid-chain-anchored membrane, (7) GPI-anchored membrane, and (8) peripheral membrane.The objective of this dataset is to classify a given query protein in a given localization. All proteins in the same subcellular location have less than 80% sequence identity. The testing protocol is based on a given subdivision, each of which is divided into a training set (3249 proteins) and a testing set (4333 proteins).

*DNA-Binding Proteins (DNA) (See [57]).* This is a dataset containing 118 DNA-binding proteins and 231 non-DNA-binding proteins with less than 35% sequence identity between each pair.

*Enzyme (ENZ) (See [58]).* This is a dataset that was created using the PDB archive and includes proteins annotated as enzymes, specifically, 381 hydrolases and 713 enzymes of different kinds.

*GO Dataset (GO) (See [58]). *This is a dataset that was extracted from the PDB archive by selecting proteins according to GO annotations. It distinguishes the biological processes “immune response” (33 proteins) and “DNA repair” (43 proteins) and the molecular functions “substrate specific transporter activity” (39 proteins) and “signal transducer activity” (53 proteins). The presence of highly similar proteins within the same class was avoided by removing sequences which had more than 30% identity.

*Human Interaction (HI) (See [59]).* This is from the positive protein-protein-interaction (PPI) dataset [59], which was downloaded from the human protein references database (HPRD, June 2007 version). This version of HPRD contains 38,788 protein-protein pairs of experimentally verified PPIs from 9,630 different human proteins. Self-interactions and duplicate interactions from the dataset were eliminated to obtain 36,630 unique positive protein-protein pairs. The benchmark negative dataset was obtained from the SWISS-PROT database (version 57.3 released on 26-May-2009) by selecting 36,480 protein couples with different cellular compartments that do not interact with each other (see [59] for details). The final dataset was constructed from the original benchmark dataset by excluding proteins having more 25% sequence identity to any of the other proteins using the PISCES program. Accordingly, the number of proteins in the positive dataset was reduced from 9,630 to 2,502, and the number of proteins in the negative dates was reduced from 2,184 to 661 for a total of 3,899 positive samples of protein pairs and 4,262 negative samples of protein pairs. This dataset is not used in all experiments because of its large size (e.g., it is not used in the first experiment to calculate the rank of the compared approaches).

*Submitochondria Locations (SL) (See [60]).* This is a dataset containing 317 proteins classified into three submitochondria locations: 131 inner membrane proteins; 41 outer membrane proteins; and 145 matrix proteins. To obtain a balance between the homologous bias and the size of the training set, no more than 40% similarity was allowed (i.e., the identity between any 2 sequences in the processed dataset had to be less than 40%).

*Virulent Datasets 1 and 2 (VI1, VI2) (See [48]).* This is a dataset containing bacterial virulent protein sequences that were retrieved from the SWISS-PROT and VFDB (an integrated and comprehensive database of virulence factors of bacterial pathogens). The two independent sets share the same training set of 1025 virulent and 1030 nonvirulent bacterial sequences. The virulent independent dataset 1 (VI1) contains 83 protein sequences, selected so that no two sequences are more than 40% similar. The virulent independent dataset 2 (VI2) contains 141 virulent and 143 nonvirulent sequences from bacterial pathogens sequences of organisms that were not represented in the training set.

*ADHESINS Dataset (AD) (See [48]).* This is a dataset containing 469 adhesins and 703 nonadhesins proteins (including several archaebacterial, viral, and yeast nonvirulent proteins). The training set contains 1025 virulent and 1030 nonvirulent bacterial sequences.

*GPCR (GP) (See [20]).* This is a dataset containing G protein-coupled receptors (GPCR) and non-GPCRs. The aim of this dataset is to identify a query protein as either GPCR or non-GPCR. None of the proteins included have ≥40% pairwise sequence identity to any other in the same subset.

*GRAM (GR) (See [61]).* This is a dataset containing gram-positive proteins that belong to five subcellular location sites: (1) cell wall, (2) cytoplasm, (3) extracellular, (4) periplasm, and (5) plasma membrane. The aim of this dataset is to classify a given query protein in a given localization. Only those proteins that have <25% sequence identity to any other in a same subcellular location were allowed to be included in the benchmark datasets. In this way, redundancy and homology bias is limited.

*Human Protein-Protein Interaction (HU) (See [62]).* This is a dataset containing a total of 1882 human protein pairs. Each pair of proteins is labeled as either an* interacting pair* or a* noninteracting pair*.

*Viral (VR) (See [63]).* This is a dataset containing proteins that belong to four classes: cytoplasm, extracellular, nucleus, and plasma membrane. The aim of this dataset is to classify a given query protein in a given localization. None of the proteins have 25% sequence identity to any other in the same subset (subcellular location). Subcellular localization of viral proteins within a host cell or virus-infected cell is very useful for studying the function of viral proteins as well as designing antiviral drugs.

*Protein Fold (PF) (See [15]).* The dataset used in this work is a subset of the database presented in [15]. The training set contains 313 proteins and the testing set contains 385 samples from 27 classes. The sequence similarities are less than 35% and 40%, respectively. The testing protocol uses the training set to build the classifier models and independently uses the testing set to evaluate performance.

*Cysteine (CY) (See [64]).* This is a dataset that was constructed to predict the state of cysteines. It contains 957 protein sequences, having a sequence identity lower than 25%. The dataset is divided into three classes: proteins that do not have disulfide bonds, which are labeled as “none” and two others that are labelled “mix” and “all” depending on whether all the cysteines have been formed into disulfide bonds or not.

*SubCell (SC).* This is a dataset containing proteins that belong to three subcellular location sites: (1) nucleus, (2) cytoplasm, and (3) extracellular. Only proteins where the PDB format is available are used. The aim is to classify a given query protein in a given localization.

The testing protocol employed in the experiments depended on the datasets. In cases where the original dataset is not divided into training and testing sets, a 10-fold cross-validation was performed (results averaged on ten experiments); otherwise the subdivision of the training and testing sets was maintained.

Three performance indicators are used in the reported results: the classification accuracy, the area under the ROC curve (AUC), and the statistical rank. The accuracy is the ratio between the number of samples correctly classified and the total number of samples. The ROC curve is a graphical plot of the sensitivity of a binary classifier versus false positives (1—specificity), as its discrimination threshold is varied. AUC [65] is a scalar measure that can be interpreted as the probability that the classifier will assign a lower score to a randomly picked positive pattern than to a randomly picked negative pattern. When a multiclass dataset is used, the one-versus-all area under ROC curve is used [66]. Since AUC is considered one of the most reliable performance indicators [67], internal comparisons are evaluated according to AUC, while accuracy is used to compare results with the literature in those cases where AUC is not reported.

The statistical rank returns the relative position of a method against other tested methods. The average rank is the most stable indicator to average performance on different datasets and is calculated using the Friedman's test (alpha = 0.05) applying the Holm post hoc procedure [68].

### 5.2. Experimental Results

The first experiment is aimed at comparing all the descriptors detailed in Section 3 and is summarized in Table 2 in terms of the statistical rank evaluated considering all the datasets (excluding HI).

In Table 3 the different methods are evaluated by their average rank, with the best descriptor for each representation highlighted. Notice that the representation method DM is not included in this table; this is because it is available only in a subset of datasets (i.e., where the PDB format is obtainable). Examining Table 3 it is clear that on average PSSM is the best representation. The other representations, however, are useful for building a strong ensemble that outperforms the results of the best stand-alone descriptors, as demonstrated by experiments reported.

The second experiment is aimed at comparing only the best descriptors found in Table 3. In Tables 4 and 5, we report the performance (in terms of AUC) of the two best descriptors for each representation (see Table 3) related to 2-class and multiclass datasets, respectively. The two best results for each dataset are highlighted. On average PP coupled with PSSM obtains the best results in most datasets, but in some problems the PP descriptor is outperformed by SAN (which is always coupled to PSSM). Some results related to HI are not reported due to the high computational costs.

The best results in the previous tables are almost always obtained with PSSM and AAS representations of proteins. Comparing the reported results of PR and WAVE with [34], where the first versions of those representations were tested, with the experiments reported here, it is clear that there is a boost in performance in PR and WAVE. See, for comparison, Table 6, where previous results obtained using these representations (SVM is the classifier) are reported.

The third experiment tests some ensemble approaches based on the fusion of some of the best descriptors, selected considering all the datasets, excluding HI. The ensembles tested in this experiment are obtained as the weighed fusion of the following methods, labelled in terms of representation (descriptor):

- FUS1: 2 × AAS(AC) + 2 × PSSM(SAN) + 4 × PSSM(PP) + PSSM(LHF_G) + PSSM(BGR) + PSSM(TGR) + SMR(PP) + SMR(BGR),
- FUS2: 2 × AAS(AC) + 2 × PSSM(SAN) + 4 × PSSM(PP) + PSSM(LHF_G) + PSSM(BGR) + PSSM(TGR) + SMR(PP) + SMR(BGR) + 2 × DM(LPQ_G) = FUS1 + 2 × DM(LPQ_G).

The results of these two ensembles are compared in Tables 7 and 8 with the best three single methods. Results related to FUS2 are reported for only a few datasets since it contains a descriptor based on the DM matrix.

The most interesting result among those reported in Tables 7 and 8 is that of our ensemble FUS1, which outperforms the other approaches in nearly all the datasets and accomplishes this performance gain without changing its weights. It is also interesting to note that even though the recent representation of SMR works rather poorly compared with PSSM and AAS, it is nonetheless useful when combined with PSSM and AAS. The other representations, WAVE, PR, and DM, are not yet useful in fusion; in our opinion, a wide survey on different texture descriptors still needs to be performed to determine which set of descriptors can boost the performance of these representations.

The forth experiment is aimed at comparing our ensembles FUS1 and FUS2 with the performance reported in the literature by other state-of-the-art approaches. Unfortunately, a fair comparison with other approaches is not always easy for the following reasons.

- Several papers use self-collected datasets and only in a few cases is the code for feature extraction available.
- Many works report results obtained on small datasets, without a clear indication of the testing protocol used; therefore, it is difficult to know whether parameter optimization was performed on the entire dataset (thereby overfitting results) or only on a training set. Overfitting is particularly dangerous in small datasets.

The comparison is much easier when considering large datasets (as with HI and MEM) or when an independent dataset separate from the training set is available (as in PF). So in the following tests we compare our results only when we are quite sure that the comparison is fair.

Tables 9 and 10 report the performance in terms of AUC and accuracy, respectively. When available we have used original source code for comparing methods. When results are extracted from the original reference paper, the best method reported in the paper is considered in the comparison. It should also be noted that in what follows we are comparing the most widely used descriptors in the literature, using whenever possible the original source code for the descriptors (not our reimplementation). To ensure fair comparison, we have also used the same testing protocol that was used in the original reference. Moreover, it should be noted that although it is the case that when small datasets are used (or when only a few datasets are tested) a jackknife approach is quite feasible, in our survey using several datasets and many descriptors, the jackknife approach becomes computationally unfeasible.

The results reported in Tables 9 and 10 are interesting not only because in this work we outperform all our previously proposed ensembles but also because we obtain state-of-the-art performances on such large datasets as HI and on such widely used benchmarks as PF and MEM. Please note that our ensemble FUS1 works well across nearly all the tested datasets, without any parameter tuning to optimize performance for a given dataset.

Considering the dataset PF, which is one of the most widely used benchmarks, FUS1 compares very well with the other approaches where features are not extracted using 3D information (for a fair comparison). The performance FUS1 is all the more valuable when considering that unlike the older approaches, ours is obtained without ad-hoc feature extractors (where the features are validated only on PF with a high risk of overfitting).

The compared approaches on PF are the following.

- Reference [15], where six kinds of features denoted by C, S, H, P, V, and Z are proposed. C is the popular amino-acid composition; the remaining five indicate the features of polarity, polarizability, normalized Van Der Waals volume, hydrophobicity, and predicted secondary structure, respectively.
- Reference [70], where the same CSHPVZ features proposed by [15] are used, but with different classifier systems.
- Reference [69], where the authors combine CSHPVZ features with bigram-coded feature (B) and spaced bigram-coded feature (SB).
- Reference [72], where the authors do the same work as [69] but improve the classifier system using the technique of data fusion.

Since the PF dataset aims at predicting the 3D structure of a protein, features extracted from 3D representations are highly useful as proven by the better performance obtained by FUS2 with respect to FUS1.

Given the results reported above, our proposed ensemble FUS1 should prove useful for practitioners and experts alike since it can form the base for building systems that are optimized for particular problems (e.g., SVM optimization and physicochemical properties selection). Obviously, it is very important that only the training data be used for physicochemical properties selection; it is not fair to choose the physicochemical properties using the entire dataset to do so. Moreover, when the ensemble is optimized for a given dataset, it is very important to consider that large descriptors work better when a large training set is available (because of the curse of dimensionality). As an example of this, we report below the performance of AAS(RC) and AAS(AC). AAS(RC) has high dimensionality and, accordingly, as seen in Table 11, works well mainly on large datasets. For this reason, it can be used in an ensemble only in the case where a large training set is available (as with MEM or HI). Notice that in HI the method AAS(RC) outperforms our best ensemble.

A similar behavior occurs with some other methods. In Table 12 we report the performance obtained by PSSM(LPQ_G). It works very poorly in some datasets but very well in others (mainly with the larger datasets).

It is clear from our experimental results that it is difficult to find an ensemble that performs the best across each of the datasets. Nonetheless, we have shown that among the several tested and proposed protein descriptors, it is always possible to find an ensemble that performs well in each type of dataset.

## 6. Conclusion

One goal in this work was to provide a survey of several state-of-the-art descriptors and some new variants starting from different protein representations. We compare the performance of these descriptors across several benchmark datasets. The results reported in this paper show that the best protein representation is PSSM, but AAS and SMR also work well. We found that no single descriptor is superior to all others across all tested datasets.

Another objective of this study was to search for a general ensemble method that could work well on different protein classification datasets. Accordingly, we performed several fusions for finding experimentally a set of descriptors based on different representations that worked well across each of the tested datasets. A couple of representations, such as WAVE and PR, were not useful in fusion. Given the results of our experiments, we concluded that a wide survey of different texture descriptors needs to be performed since different descriptors contain different information that might boost performance when combined with others.

Our major contribution is to propose an ensemble of descriptors/classifiers for sequence-based protein classification that not only works well across several datasets but also, in some cases, proves superior to the state-of-the-art. Unlike other papers that develop a web server, we share almost all the MATLAB codes used in the proposed approaches. Our proposed ensemble could be considered a baseline system for developing an ad-hoc system for a given problem. Issues to consider when optimizing such a base system for a given dataset were also discussed. For instance, the size of datasets seems to play a role in the choice of protein representation, with some descriptors showing stronger performance on large datasets. In particular, approaches that use a high dimensional representation (e.g., RC) requires larger datasets in order to avoid the curse of dimensionality.

To further improve the performance of our methods, we plan, in the future, on testing more classification approaches. We are particularly interested in investigating ensembles made with AdaBoost and Rotation forest [74] classifiers. The main drawback using these ensemble methods is that they require more computational power than SVM, the classifier used in this work. Although this would not be a problem for the testing phase, it would be a drawback in the training phase if we want to compare a number of different descriptors across several (preferably large) datasets.

## Figures and Tables

**Table 1:** Summarized description of the datasets (if available, the number of training and independent samples is given in column “number of samples”). The column BKB reports whether it is possible from the dataset to obtain the PDB of the proteins for extracting the backbone structure.

Name	Short name	Number of samples	Number of classes	Protocol	BKB
Membrane subcellular	MEM	3249 + 4333	8	Independent training and testing sets	NO
Human pairs	HU	1882	2	10-fold cross validation	NO
Protein fold	PF	698	27	Independent training and testing sets	YES
GPCR	GP	730	2	10-fold cross validation	NO
GRAM	GR	452	5	10-fold cross validation	NO
Viral	VR	112	4	10-fold cross validation	NO
Cysteines	CY	957	3	10-fold cross validation	YES
SubCell	SC	121	3	10-fold cross validation	YES
DNA-binding proteins	DNA	349	2	10-fold cross validation	YES
Enzyme	ENZ	1094	6	10-fold cross validation	YES
GO dataset	GO	168	4	10-fold cross validation	YES
Human interaction	HI	8161	2	10-fold cross validation	NO
Submitochondria locations	SL	317	3	10-fold cross validation	NO
Virulent independent set 1	VI1	2055 + 83	2	Independent training and testing sets	NO
Virulent independent set 2	VI2	2055 + 284	2	Independent training and testing sets	NO
Adhesins	AD	2055 + 1172	2	Independent training and testing sets	NO

**Table 2:** Summary of the descriptors (short names are defined in Sections 3 and 4).

Descriptors		
Protein representation	Descriptor	Size
AAS	AS	20
	2G	400
	QRC^*d*^	1200
	AC^*d*^	40
	P2G^*d*^	800
	AA^*d*^	65
	GE	480
	NG	400, 225, 512, 125, 64
	SAC	20
	DW^*d*^	52

PSSM/SMR PR/WAVE (ensembles of 25 PR^*d*^/WAVE^*d*^) DM	AB	400
	SAN	400
	SA	400
	AM	300
	PP	320
	SVD	Depends on the input representation
	DCT	400
	LHF_G	176
	LPQ_G	512
	LHF_L	528
	LPQ_L	1536
	BGR	400
	TGR	8000

**Table 3:** Comparison among the different feature extractors in terms of the statistical rank on the different datasets. The 2 best descriptors for each representation are in boldface.

Descriptors		Rank
Protein representation	Descriptor	
AAS	AS	23.42
	2G	27.25
	QRC^*d*^	21.54
	AC^*d*^	**11.52**
	P2G^*d*^	39.78
	AA^*d*^	**21.36**
	GE	30.24
	NG	27.85
	SAC	23.45
	DW^*d*^	29.48

PSSM	AB	15.25
	SAN	**7.25**
	SA	13.20
	AM	20.50
	PP	**5.02**
	SVD	39.56
	DCT	28.56
	LHF_G	24.10
	LPQ_G	14.87
	LHF_L	31.81
	LPQ_L	26.72
	BGR	12.44
	TGR	15.68

SMR	AB	28.78
	SAN	24.80
	SA	24.82
	AM	40.52
	PP	**12.50**
	SVD	29.20
	DCT	32.45
	LHF_G	**17.02**
	LPQ_G	17.22
	LHF_L	26.24
	LPQ_L	31.24
	BGR	19.86
	TGR	23.24

PR (ensemble of 25 PR^*d*^)	SVD	**38.25**
	DCT	**37.85**
	LHF_G	41.25
	LPQ_G	38.38
	LHF_L	44.02
	LPQ_L	38.48

WAVE (ensemble of 25 WAVE^*d*^)	SVD	40.25
	DCT	47.00
	LHF_G	**38.95**
	LPQ_G	**34.01**
	LHF_L	41.10
	LPQ_L	40.20

**Table 4:** Comparison in terms of AUC in 2 class problems.

AUC		Datasets						
Protein representation	Descriptor	DNA	HU	HI	GP	AD	VI1	VI2
AAS	AC^*d*^	92.6	71.8	**96.4**	99.1	80.9	**90.0**	76.5
	AA^*d*^	90.6	68.3	—	98.8	78.9	**89.2**	75.6

PSSM	PP	**95.5**	**81.3**	94.8	**99.8**	**87.7**	86.2	**87.3**
	SAN	**95.2**	**76.4**	**95.7**	**99.7**	**82.7**	87.3	**85.7**

SMR	PP	92.9	73.8	—	99.5	79.8	88.5	76.0
	LHF_G	89.3	69.0	—	99.3	81.6	83.4	71.1

PR	SVD	79.6	74.2	—	98.0	72.3	59.1	73.3
	DCT	83.4	67.7	—	95.9	73.4	68.4	63.0

WAVE	LPQ_G	83.1	68.6	—	98.5	74.0	67.4	67.6
	LHF_G	77.7	68.6	—	97.8	68.9	65.1	60.8

**Table 5:** Comparison in terms of AUC in multiclass problems.

AUC		Datasets								
Protein representation	Descriptor	MEM	PF	ENZ	GR	VR	SL	CY	GO	SC
AAS	AC^*d*^	93.6	84.8	66.7	92.7	**81.8**	93.2	78.4	70.0	67.6
	AA^*d*^	90.4	84.2	63.7	92.6	72.2	91.1	76.5	69.5	65.5

PSSM	PP	**96.8**	**93.1**	**78.0**	80.8	**81.8**	**95.7**	**79.4**	**84.5**	**70.3**
	SAN	95.5	**87.7**	**71.2**	**93.0**	72.0	**94.1**	**81.8**	**78.6**	**73.9**

SMR	PP	94.2	85.9	66.2	**92.8**	76.9	92.2	78.7	69.0	66.2
	LHF_G	**96.2**	87.6	65.6	91.3	**82.4**	89.5	78.2	72.4	62.9

PR	SVD	94.4	83.5	59.4	80.8	76.0	85.4	73.5	59.7	60.3
	DCT	91.7	79.5	60.8	82.6	74.2	83.9	71.7	65.3	64.2

WAVE	LPQ_G	94.2	87.2	63.2	82.7	79.2	83.4	68.1	65.7	58.1
	LHF_G	92.7	86.2	61.5	80.3	80.6	81.0	66.6	65.2	57.0

**Table 6:** Comparisons with previous versions of WAVE and PR.

AUC		Dataset		
Protein representation	Descriptor	HU	GP	AD
WAVE	Best in [34]	66.1	96.6	67.1
PR	Best in [34]	62.8	87.8	57.5
WAVE	LPQ_G	68.6	**98.5**	72.3
PR	SVD	**74.2**	98.0	**74.0**

**Table 7:** Comparison among ensembles and best stand-alone descriptors in terms of AUC in 2 class problems.

AUC	Datasets						
Protein representation	DNA	HU	HI	GP	AD	VI1	VI2
PSSM(PP)	95.5	81.2	94.8	99.8	87.7	86.2	87.2
PSSM(SAN)	95.2	76.4	95.7	99.7	82.7	87.3	85.7
AAS(AC)	92.6	71.8	95.9	99.1	80.9	90.0	76.4
FUS1	97.2	**82.0**	**98.4**	**99.9**	**88.2**	**89.0**	**88.7**
FUS2	**97.3**	—	—	—	—	—	—

**Table 8:** Comparison among ensembles and best stand-alone descriptors in terms of AUC in multiclass problems.

AUC	Datasets								
Protein representation	MEM	PF	ENZ	GR	VR	SL	CY	GO	SC
PSSM(PP)	96.8	93.1	78.0	80.8	81.8	95.7	79.4	**84.5**	70.3
PSSM(SAN)	95.5	87.7	71.1	**93.0**	72.0	94.1	81.8	78.6	73.4
AAS(AC)	93.6	84.8	66.7	92.7	81.8	93.2	78.4	70.0	67.6
FUS1	**97.1**	92.7	**80.2**	92.3	**84.7**	**96.7**	**84.5**	83.8	75.3
FUS2	—	**95.9**	80.1	—	—	—	84.3	82.8	**76.4**

**Table 9:** Comparison with the state-of-the-art using AUC as performance indicator.

AUC	Datasets													
Methods	HU	PF	GP	GR	VR	DNA	ENZ	MEM	GO	SL	HI	AD	VI1	VI2
[48]												77.0	87.0	83.4
[58]						93.3	72.5		50.0					
[12]	72.5		99.7	**94.7**	82.5			96.0				82.9	86.1	76.0
[59]											98.2			
[34]												81.6	91.2	84.1
[28]						95.9	79.4	96.8		93.8	98.0		87.1	87.9
FUS1	**82.0**	92.7	**99.9**	92.3	**84.7**	**97.2**	**80.2**	**97.1**	**83.8**	**96.7**	**98.4**	**88.2**	**89.0**	**88.7**

**Table 10:** Comparison with the state-of-the-art using accuracy as performance indicator.

Accuracy	Datasets													
Methods	HU	PF	GP	GR	VR	DNA	ENZ	MEM	GO	SL	HI	AD	VI1	VI2
[15]		56.50												
[69]		65.50												
[70]		58.18												
[40]		61.04												
[71]	70.0													
[72]		69.60												
[3–5]								91.6						
[20]			91.6											
[61]				84.1										
[1]								92.7						
[73]								92.6						
[12]	70.0		98.1	84.4	78.6			91.5						
[28]							56.2	94.1	59.4	85.8	93.1		85.5	81.7
FUS1	**75.0**	68.6	**99.2**	87.9	**76.2**	**93.7**	**56.9**	94.3	64.3	**87.0**	93.9	**78.0**	**84.3**	**83.5**
FUS2		74.6				**94.6**	**57.1**		63.0					

**Table 11:** Comparison between AAS(RC) and AAS(AC).

AUC	Datasets															
Methods	HU	PF	GP	GR	VR	DNA	ENZ	MEM	GO	SL	HI	AD	VI1	VI2	CY	SC
AAS(RC)	70.3	**87.2**	98.9	90.0	69.0	86.2	64.5	**95.9**	68.3	87.8	**98.9**	**81.1**	89.2	75.9	77.6	62.4
AAS(AC)	**71.8**	84.8	**99.1**	**92.7**	**81.8**	**92.6**	**66.7**	93.6	**70.0**	**93.2**	95.9	80.9	**90.0**	**76.4**	**78.4**	**67.6**

**Table 12:** Comparison among ensembles and best stand-alone descriptors in terms of AUC.

AUC	Datasets															
Methods	HU	PF	GP	GR	VR	DNA	ENZ	MEM	GO	SL	HI	AD	VI1	VI2	CY	SC
PSSM(PP)	**81.2**	**93.1**	99.8	80.8	**81.8**	**95.5**	**78.0**	**96.8**	**84.5**	**95.7**	94.8	**87.7**	86.2	**87.2**	79.4	70.3
PSSM(SAN)	76.4	87.7	99.7	**93.0**	72.0	95.2	71.1	95.5	78.6	94.1	95.7	82.7	**87.3**	85.7	**81.8**	**73.4**
PSSM(LPQ_G)	72.0	89.5	**99.9**	82.3	77.7	89.5	66.2	93.6	73.0	93.7	**97.6**	86.8	82.3	83.9	70.3	61.6
